# *UGT1A1* Gene Mutation due to Crigler-Najjar Syndrome in Iranian Patients: Identification of a Novel Mutation

**DOI:** 10.1155/2013/342371

**Published:** 2013-10-28

**Authors:** Javad Mohammadi Asl, Mohammad Amin Tabatabaiefar, Hamid Galehdari, Kourosh Riahi, Mohammad Hosein Masbi, Zohre Zargar Shoshtari, Fakher Rahim

**Affiliations:** ^1^Department of Medical Genetics, Faculty of Medicine, Ahvaz Jundishapur University of Medical Sciences, Ahvaz, Iran; ^2^Genetic Department, Faculty of Science, Shahid Chamran Univerity, Ahvaz, Iran; ^3^Pediatric Department, Ahvaz Jundishapur University of Medical Sciences, Golestan Hospital, Golestan, Ahvaz, Iran; ^4^Noor Genetic Diagnostic Laboratory, Ahvaz, Iran; ^5^Toxicology Research Center, Ahvaz Jundishapur University of Medical Sciences, Ahvaz, Iran

## Abstract

Crigler-Najjar syndrome (CNS) type I and type II are inherited as autosomal recessive conditions that are caused by mutations in the *UGT1A1* gene. We present the analysis of *UGT1A1* gene in 12 individuals from three different families. This analysis allowed us to identify one novel mutation, which was not previously described. In this study, three families with clinically diagnosed CNS referred from Khuzestan province, southwest Iran, were screened. After signing the informed consent, peripheral blood samples from the patients and their parents were collected in EDTA-containing tube followed by DNA extraction using a routine phenol-chloroform method. All five coding exons and the flanking intronic regions of the bilirubin-UGT were amplified by polymerase chain reaction (PCR) followed by DNA sequencing by Sanger method. From the first family, a 9-month-old boy was homozygous for a deletion mutation of two adjacent nucleotides including one adenosine (A) and one glutamine (G) between nucleotides 238 and 239 in exon 1 (c.238_240 del AG). In the second family, there were two affected individuals, an 11-year-old girl and a fetus, found to be homozygous for the same mutation. The third family showed a mutation at nucleotide 479 in exon 1 (Val160Glu) that has been reported previously. Molecular analysis can significantly help confirm the diagnosis of CNS, without any need for the liver biopsy, and may help the therapeutic management by ruling out more harmful causes of hyperbilirubinemia.

## 1. Introduction

Crigler-Najjar syndrome (CNS) (MIM nos. 218800, 606785) type I and type II are inherited as autosomal recessive conditions that are caused by mutations in the *UGT1A1 *gene (*UGT1A1*; MIM no. 191740) [1–6]. Type I is characterized by almost complete absence of UGT1A1 enzyme activity, in which the affected individuals are refractory to phenobarbital treatment, while type II is a less severe form of deficiency [6, 7]. Patients with CNS are at permanent risk of developing severe neurologic complications such as hearing problems, mental retardation, and choreoathetosis due to severe unconjugated hyperbilirubinemia [8]. The high performance liquid chromatography (HPLC) analysis of liver enzyme assay is the conclusive diagnosis of this syndrome [9]. The *UGT1A1 *gene comprises five consecutive exons located at the 3′end of the *UGT1A *locus on chromosome 2q37.

Mutations in the *UGT1A1 *gene occur in any of the five exons, which either completely (CNS-I) or partially (CNS-II) inactivate this enzyme and result in a truncated protein. Here, we present the analysis of *UGT1A1 *gene in 12 unrelated individuals from three different families.

## 2. Subjects and Methods

### 2.1. Cases History

*Family A.* The patient was a 9-month-old boy who has had jaundice 72 h after birth continuously, with high total and direct bilirubin values (Table 2). His first blood examination revealed a normal hemoglobin level (12.5 g/dL), RBC (4.89 M/*μ*L), MCV (74.8 fL), MCH (25.6 pg), MCHC (34.2 g/dL), and platelet count (299 k/*μ*L). Both parents (22-year-old father and 21-year-old mother) were educated with a history of mild jaundice, consanguinity, and mental disorders in their relatives. During a seven-day course of phototherapy treatment without phenobarbital, the serum bilirubin has dropped to 23 mg/dL. There was no evidence of neurotoxicity. He was transferred to our department for further diagnosis. 

*Family B.* A pregnant woman with a fetus was referred to our department at gestational age of 18 weeks for amino synthesis. Both parents (28-year-old father and 26-year-old mother) were educated with no history of consanguinity and mental disorders in their relatives. Their other child was an 11-year-old girl with jaundice. Her first blood examination revealed hemoglobin (14.2 g/dL), RBC (5.60 M/*μ*L), MCV (82.0 fL), MCH (25.6 pg), MCHC (31.2 g/dL), and platelet (217 k/*μ*L). Her family history revealed that her parents had mild jaundice (Table 2). Her birth weight was 3500 g following normal pregnancy and uneventful cesarean section. This girl developed clinical jaundice by 4 days of age with total and direct bilirubin of 26 mg/dL and 1.5 mg/dL, respectively. Her hyperbilirubinemia responded to a 12-day course of phototherapy without phenobarbital, and total bilirubin was reduced to 13 mg/dL. She was discharged under phenobarbital therapy, but her hyperbilirubinemia rebounded quickly, and she was admitted again with a total bilirubin of 29 mg/dL and was transferred to our department for further diagnosis. 

*Family C.* A consanguine couple (29-year-old male and 26-year-old female) was referred to our department for premarital genetic counseling and screening of CNS. The patient was a 29-year-old male who has had jaundice, with a total bilirubin of 8.4 mg/dL, direct bilirubin of 1.8 mg/dL, and blood examination results of hemoglobin (15.2 g/dL), RBC (4.67 M/*μ*L), MCV (79.1 fL), MCH (24.2 pg), MCHC (32.8 g/dL), and platelet count (287 k/*μ*L). The man had a family history of CNS. His parents (52-year-old father and 49-year-old mother) were not educated with a history of slight elevation in bilirubin (Table 2) and consanguinity and no history of mental disorders in their relatives. Their other child was an 11-year-old boy with jaundice, with a total and direct bilirubin of 26 mg/dL and 0.56 mg/dL, respectively. The woman was normal with no history of CNS in her family and relatives.

### 2.2. Molecular Genetic Analysis

#### 2.2.1. DNA Extraction and Nucleotide-Sequence Analysis

Following the approval of the local ethics committee of Ahvaz Jundishapur University of Medical Sciences (AJUMS) and taking a written informed consent from parents, peripheral blood samples were obtained. Genomic DNA was isolated from peripheral blood of the patients, their parents, and several normal individuals using a routine phenol-chloroform method. All five coding exons and the flanking intronic regions of the bilirubin-UGT were amplified by polymerase chain reaction (PCR), according to Bosma et al., method [10–12]. Then, all PCR products were sequenced using designed primers (Primer 3.0 web based software, University of Massachusetts Medical School, USA) (Table 1).

The reaction mixture (30 *μ*L) contains 2 *μ*L of genomic DNA, 1 *μ*L of each upstream and downstream primer (diluted to 10 *μ*mol/L), 11 *μ*L of ddH_2_O, 15 *μ*L of 2 × Taq PCR Master Mix (Qiagen Inc.) were used, as described by Wang et al., recently. In brief, PCR was implemented by denaturation at 95°C for 3 min, followed by 40 thermal cycles composed of 94°C for 30 sec, 55°C for 30 sec, and 72°C for 45 sec for each. Furthermore, the amplified products were isolated by electrophoresis on a 1% agarose gel and then purified using the QIAamp purification kit (Qiagen Inc.), and the nucleotide sequence was determined by direct sequencing using an ABI 310 automatic sequencer according to the manufacturer's instructions (Applied Biosystem, Foster City, CA, USA).

## 3. Results

**Figure 1:**
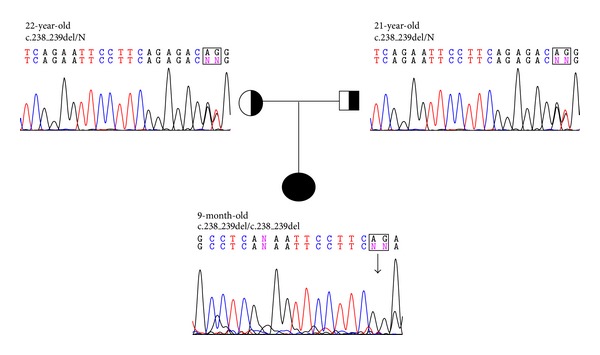
The family pedigree and sequencing chromatograms of the *UGT1A1* gene showing a homozygous two bp deletion (c.238_240delAG, exon 1) in the proband from family A and a heterozygous deletion in the father and the mother of each case; appearance of her chromatogram is comparable to that of a normal control; N, normal.

**Figure 2:**
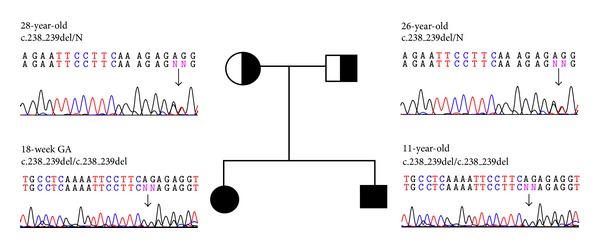
The family pedigree and sequencing chromatograms of the *UGT1A1* gene showing a homozygous two bp deletion (c.238_240delAG, exon 1) in the proband from family B and a heterozygous deletion in the father and the mother of each case; appearance of her chromatogram is comparable to that of a normal control; N, normal.

From family A, a 9-month-old boy with clinical CNS was homozygous for a 2-bp deletion mutation causing a change of the coding exons with a frameshift. The deletion of one adenosine (A) and one glutamine (G) between nucleotides 238 and 240 in exon 1 (Figure 1) modified the nucleotide of codons 238, 239, and 240 (Table 2). Sequencing showed the homozygous mutation, which had not been reported so far. The evaluation of parents revealed the heterozygous pattern of the same mutation in both (Figure 1 and Table 2). In family B, there were two individuals (B and C), an 11-year-old girl and 18-week old fetus. Patient B, a girl with clinical CNS, and patient C, a fetus, were homozygous for the same deletion as patient A (Figure 2). Both parents were also heterozygous for the same mutation (Figure 1 and Table 2).

In family C, there were two boys and their parents with clinical CNS. From these four subjects, three were heterozygous and one was homozygous for substitution mutation. Polymerase chain reaction and sequence analysis of the *UGT1A1 *gene revealed a T>A mutation at nucleotide 479 in exon 1 (Val160Glu) that has been reported previously (Table 2) [2].

## 4. Discussion

Mutations in the *UGT1A1* gene were previously shown to cause CNS. Here we describe a unique etiology of CNS in three affected families, which was analyzed using direct sequencing of whole coding sequences and splice sites of the *UGT1A1* gene. Overall seven heterozygous and four homozygous cases were observed. The deletion mutation in exon 1 may alter the structure of a protein called UDPGT due to the frameshift. The enzymatic activity of the resulting protein may reduce because it has amino acids that are different in addition to the premature translation at the codon of interest. 

CNS is a rare entity with only few hundred cases reported since the first report in 1952 [13]. The incidence of CNS is estimated in a range of 0.6–1.0 per million live births [14]. Although the clinical characteristics of our patients were consistent with prior reported cases, the observed mutation was novel and different. Bilirubin encephalopathy due to CNS may occur without well-timed treatment [15]. 

Careful phenotypic analysis of patients who have disorders caused by UDPGT can be informative for identification of parent-of-origin differences in gene expression. Consistent with this, the mother and the father, who were heterozygous for the c.238_240del, had normal or slightly elevated serum bilirubin. It can be deduced, therefore, that in the case, homozygous for the novel deletion at the exon 1, was required for the expression of the jaundiced phenotype. Although over 65 different genetic mutations in the coding region of *UGT1A1 *gene have been described to cause CNS, [16, 17] the novel deletion reported here was found to inactivate the enzyme. 

The characterization of a genetic defect which is present in the large majority of subjects with CNS makes a genetic test for this disorder feasible, if not practical. Moreover, knowing the molecular genetics of CN has clinical consequences that are not limited to the field of familial unconjugated hyperbilirubinemias. In all these instances, direct recognition of the role of CNS may help in the diagnostic and therapeutic management of the patient, by ruling out more harmful causes of hyperbilirubinemia. 

In conclusion, we report a novel deletion within exon 1 of *UGT1A1 *gene, causing the jaundiced phenotype, thereby inactivating the enzyme. Though *UGT1A1* gene is expressed specifically in the liver, we made an attempt to define the diagnosis and the molecular mechanism of *UGT1A1* deficiency by a noninvasive molecular approach, not demanding a liver biopsy. This method not only is valuable in starting the clinical diagnosis and given that genetic counseling, but also could help the novel gene therapy approaches that involve the knowledge of the specific genetic lesion in a patient.

## Figures and Tables

**Table 1:** *UGT1A1* gene primers used in PCR and sequencing.

Region	Primer	Primer sequence (5′→3′)
Pro	GCDH_pro_1F	GCTACTTGGAGGCTGAGGTG
	GCDH_pro_1R	TGAGGCTTTTCATTGGTTCC
Pro	GCDH_pro_2F	AACTGACCAACCAGGAGTGG
	GCDH_pro_2R	CTCTCAGGGCCATGCTCTCT
Exon 1	GCDH9_ex1F	GATTTCCACAGGCAAGGCTA
	GCDH_ex1R	CTCCTTCCCTCCACACTCC
Exon 2	GCDH_ex2F	GGAACCAATGAAAAGCCTCA
	GCDH_ex2R	GCGACCAGAGGTCCTTACAC
Exon 3	GCDH_ex3F	TCGCTCTGAGAGAGCATGG
	GCDH_ex3R	CTGCCTTAGTGCCTCTGACC
Exon 4	GCDH_ex4F	CAGGGTCAGAGGCACTAAGG
	GCDH_ex4R	GAAAAACTGCAAAGGGACCA
Exon 5	GCDH_ex5F	AGATCCTCATCAGGGACACCT
	GCDH_ex5R	CCAGCTACTAGAGGGGTGGAG
Exon 6	GCDH_ex6F	GCCACAATTTCCCAGTCTGT
	GCDH_ex6R	CAGAATAGGCTCAGGGGACA
Exon 7	GCDH_ex7F	CATATTGGTCAGGCTGGATTTT
	GCDH_ex7R	CACTACAAACAGATCGGCCATA
Exon 8	GCDH_ex8F	TGGGACCAAGACCTGGTAAG
	GCDH_ex8R	CCGGCTGAGTAAGAATCACC
Exon 9	GCDH_ex9F	TTCCCTGCTTCAGAGTTGGT
	GCDH_ex9R	AGGACGTCACTGGTCATTCC
Exon 10	GCDH_ex10F	CAGTGACGTCCTTCTGAGCA
	GCDH_ex10R	AGGACAAGAGGGACAGCAGA
Exon 11	GCDH_ex11F	GGACCTGAACCTTCTGCTGT
	GCDH_ex11R	CTCCAGATGAGGGACAGAGG
Exon 12	GCDH_ex12_1F	TGGCCTCACAGTCTTCTCCT
	GCDH_ex12_1R	CTCCCTCACTCTGCTCCAAC
Exon 12	GCDH_ex12_2F	GGATGGAGTGGGAAGTGAGA
	GCDH_ex12_2R	CCAGACCACTCTAGGGGAAA

**Table 2:** Mutations identified in patients with Crigler-Najjar syndrome (CNS).

Patients	Reported consanguinity	Bilirubin (mg/dL)		Age/sex (F: female; M: male)	Mutation	Polymorphisms	Reference
		Total	Direct				
Family A							
CNS-1	Yes	31.2	1.79	9-month-old/M	c.238_240del/c.238_240del	Del(AG)	Novel
CNS-2	Yes	1.53	0.97	22-year-old/M	c.238_240del/N	Del(AG)	Novel
CNS-3	Yes	2.1	1.22	21-year-old/F	c.238_240del/N	Del(AG)	Novel

Family B							
CNS-4	No	—	—	18 weeks GA/—	c.238_240del/ c.238_240del	Del(AG)	Novel
CNS-5	No	3.6	1.78	28-year-old/M	c.238_240del/N	Del(AG)	Novel
CNS-6	No	1.69	1.01	26-year-old/F	c.238_240del/N	Del(AG)	Novel
CNS-7	No	26	1.5	11-year-old/F	c.238_240del/c.238_240del	Del(AG)	Novel

Family C							
CNS-8	Yes	26	0.56	11-year-old/M	c.479T>A/c.479T>A	T>A	Huang et al., 2006 [2]
CNS-9	Yes	1.48	0.99	49-year-old/F	c.479T>A/N	T>A	Huang et al., 2006 [2]
CNS-10	Yes	2.61	1.13	52-year-old/M	c.479T>A/N	T>A	Huang et al., 2006 [2]
CNS-11	Yes	8.4	1.8	29-year-old/M	c.479T>A/N	T>A	Huang et al., 2006 [2]
CNS-12	Yes	0.8	0.3	28-year-old/F	N/N	T>A	Huang et al., 2006 [2]
